# Body shape and pants size as surrogate measures of obesity among males in epidemiologic studies

**DOI:** 10.1016/j.pmedr.2020.101167

**Published:** 2020-07-13

**Authors:** Eric Vallières, Marie-Hélène Roy-Gagnon, Marie-Élise Parent

**Affiliations:** a Epidemiology and Biostatistics Unit, Centre Armand-Frappier Santé Biotechnologie, Institut National de la Recherche Scientifique, University of Quebec, 531 Boul. Des Prairies, Laval, QC H7V 1B7, Canada; b School of Public Health, Department of Social and Preventive Medicine, University of Montreal, 7101 Avenue du Parc, Montreal, QC H3N 1X9, Canada; c School of Epidemiology and Public Health, University of Ottawa, 600 Peter Morand Crescent, Ottawa, ON K1G 5Z3, Canada; d University of Montreal Hospital Research Centre, 900 Saint-Denis, Tour Viger, Pavillon R, Montreal, QC H2X 0A9, Canada

**Keywords:** Obesity, Abdominal, Weight, Body mass index, Waist circumference, Hip circumference, Waist-hip ratio, Silhouette, Pants size, Prediction model

## Abstract

This study aimed at characterizing anthropometric indicators that can be used as alternatives to measurements for assessing overall obesity over adulthood and abdominal obesity among men. We used data from a population-based case-control study of prostate cancer conducted in Montreal, Canada in 2005–2012. It included men aged ≤ 75 years, 1872 of which were newly diagnosed with prostate cancer, and 1918 others randomly selected from the electoral list. In-person interviews elicited reports of height as well as of weight, pants size and Stunkard’s silhouette at 5 time points over adulthood, i.e., for the ages of 20, 40, 50 and 60 years, if applicable, and at the time of interview. Waist and hip circumferences were measured by interviewers following a validated protocol. Analyses were conducted on the overall sample of 3790 subjects, after having confirmed that results did not differ according to disease status. Stunkard’s silhouette scale proved to be an easy-to-administer tool that reflects well reported body mass index, either recently or decades in the past among adult males. It was discriminatory enough to classify individuals according to commonly-used obesity categories. We observed that a model including age, reported pants size, silhouette and weight can reasonably predict current abdominal obesity. In conclusion, alternative anthropometric indicators can serve as valuable means to assess overall and abdominal obesity when measurements cannot be envisaged in the context of epidemiological studies.

## Highlights

• Alternative anthropometric indicators reflect overall and abdominal obesity in males.
• Abdominal obesity is predicted using age, pants size, Stunkard’s silhouette & weight.
• Stunkard’s silhouette scale reflects well body mass index recently and in the past.

## 1 Introduction

Obesity, widely recognized as a risk factor for several health conditions (Dis Ineke et al., 2009, Song et al., 2016, Nunez et al., 2017, World Cancer Research Fund/American Institute for Cancer Research, 2018), continues to be of major research interest. Both overall (Samanic et al., 2006, Bhaskaran et al., 2014) and abdominal (Lavalette et al., 2018, Rhee et al., 2018) obesity have been associated with health outcomes. Overall obesity, indicated by a higher body mass index (BMI), and abdominal obesity, reflected by a higher waist circumference or waist-hip ratio (WHR), are associated with insulin resistance, dyslipidemia, and systemic inflammation, which are involved in the pathogenesis of cardiovascular disease, metabolic syndrome and certain cancers (De Pergola and Silvestris, 2013). The two main types of adipose tissue, subcutaneous (fat tissue between the skin and muscle) and visceral (within the main cavities of the body) differ in their metabolic activity, with abdominal visceral adipocytes being metabolically more active (Calle and Kaaks, 2004).

Body size can be assessed in different ways. Weight and BMI are the most commonly used indicators of overall obesity (Afshin et al., 2017, World Cancer Research Fund/American Institute for Cancer Research, 2018). While direct measurement by trained personnel would be preferable (Gorber et al., 2007, Okamoto et al., 2017), self-reports of weight and height are commonly used in epidemiological studies given their practicality. Several investigations document a high correlation between measured and self-reported values (Bulik et al., 2001, Spencer et al., 2004, Gorber et al., 2007, Elgar and Stewart, 2008, Lim et al., 2012, Poston et al., 2014, Dratva et al., 2016). However, of particular interest here, some studies observed that men tend to overestimate their height (by 0.3–1.6 cm, on average) (Nyholm et al., 2007, Pasalich et al., 2014, Poston et al., 2014, Okamoto et al., 2017). The accuracy of self-reported measures also tends to decrease with increasing age (Nyholm et al., 2007, Elgar and Stewart, 2008) and longer recall periods (Stevens et al., 1990, Must et al., 1993, de Fine Olivarius et al., 1997, Gunnell et al., 2000).

**Fig. 1:**
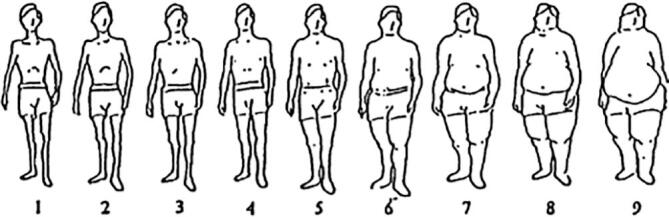
Body silhouettes scale (as first proposed by Stunkard, Sorensen et al.)(Stunkard et al., 1983).

Somatotypes scales have been developed as an alternative to traditional anthropometric indicators (Sorensen et al., 1983, Stunkard et al., 1983, Thompson and Gray, 1995, Gardner et al., 1999). These, first introduced in 1983 by Stunkard, Sorensen *et al* (Stunkard et al., 1983) as an easy-to-administer measure of body size, depict nine different body silhouettes for men and women, ordered from very lean to very obese (Fig. 1). They have been used in numerous observational studies to describe oneself (Er et al., 2014, Moller et al., 2015, Zhang et al., 2015), a family member (Sorensen and Stunkard, 1993, Napolitano et al., 2010), or how one would like to look (Johnson et al., 2012). Silhouette pictograms also serve as a useful visual guide to help remember and report body size at different times in life. They have been subjected to validity studies (Sorensen et al., 1983, Mueller et al., 1985, Must et al., 1993, Bulik et al., 2001), primarily through comparisons with BMI. While often based on small samples, correlations between measured weight (or BMI) and reported silhouettes ranged from 0.60 to 0.87 (Mueller et al., 1985, Must et al., 1993, Dratva et al., 2016, Song et al., 2016, Lønnebotn et al., 2018). Good sensitivity and specificity were observed. Moreover, silhouettes have been found to be good predictors of mortality and health outcomes, including cardiovascular diseases (Garg et al., 2015, Song et al., 2016).

The waist circumference and the WHR are commonly used to assess abdominal obesity as they reflect intra-abdominal fat accumulation (Hughes et al., 2009). However, most people cannot report their waist and hip circumferences with accuracy, so measurement using a predetermined protocol is often necessary. Validity studies have indeed shown that the waist circumference is seldom known, and often underreported (Battram et al., 2011), while its self-measurement tends toward an underestimation (Spencer et al., 2004, Lim et al., 2012, Okamoto et al., 2017). Men overrate their self-measured waist circumference by 0.6–0.8 cm, on average (Pasalich et al., 2014). Despite these shortcomings, waist circumference mainly, but also WHR, are good indicators of fat distribution (Chan, 2003, Ketel et al., 2007), and good predictors of cardiovascular disease and mortality (Dalton et al., 2003, de Koning et al., 2007, Jacobs, 2010). Waist circumference is the main indicator of visceral obesity in the definition of metabolic syndrome (Blanc-Lapierre et al., 2015).

The literature remains scant on alternative approaches to assess abdominal obesity. It has been proposed that reported clothing size, more often pants size for men, would be a good surrogate indicator. A handful of studies investigating this relationship among men found a good correlation between waist circumference and pants size (Han et al., 2005, Hughes et al., 2009, Battram et al., 2011, Moy et al., 2018). However, these mostly relied on self-reported rather than professionally measured waist circumference or were based on few subjects. Clothing size has been associated with cancer risk and other morbidities (Hughes et al., 2011, Nafiu and Burke, 2013, Moy et al., 2018).

In the current study, focusing on adult males, we describe how two alternative anthropometric indicators of overall and abdominal obesity relate to those traditionally used. We examine first how Stunkard’s silhouettes relate to reported weight and BMI across adulthood, and then how pants size reflects measured waist circumference and WHR. Novel prediction models using alternative anthropometric indicators are proposed to improve on the assessment of waist circumference and WHR.

## 2 Methods

We used data from the population-based case-control study PROtEuS (Prostate Cancer & Environment Study), conducted in Montreal, Canada in 2005–2012, and described previously (Spence et al., 2014, Blanc-Lapierre et al., 2015). PROtEuS aims at assessing the role of environmental and lifestyle factors in prostate cancer risk. Eligible subjects were men, younger than 76 years of age at diagnosis or selection, Montreal residents, registered on Quebec’s electoral list and Canadian citizens.

Cases were patients newly diagnosed with prostate cancer actively ascertained through pathology departments across French hospitals in the Montreal area. This covered over 80% of all prostate cancers diagnosed in Montreal during the study period. Concomitantly, controls were randomly selected from the electoral list of men residing in the same districts as cases and frequency-matched to cases (±5-years). Overall, 1933 cases and 1994 controls participated. Participation rates among eligible subjects were 79% for cases and 56% for controls. A comparison of census-based sociodemographic characteristics of participants and nonparticipants showed little differences between the two groups, alleviating possible concerns of selection bias in the original case-control study. Proxy respondents (<4%) were excluded from analyses.

During face-to-face interviews, subjects provided details on several anthropometric factors. For each of the ages of 20, 40, 50 and 60 years, and for the time of interview, subjects reported their weight (kilograms or pounds), pants size (US chart, see Supplementary Table A.1 for an international conversion chart), and which of Stunkard’s silhouette (from 1 to 9) best described them. When asked about the latter, subjects were invited to recall pictures of themselves at significant life events (anniversaries, marriage, etc.). Reported height (in cm or inches) was elicited for the age of 20 years and at interview. Waist and hip circumferences were measured by interviewers following a validated protocol (2.5 cm above the umbilicus for the waist, maximum for hip circumference) (World Health Organization 2011). The study was approved by the ethics boards of all participating institutions (Supplementary Table A.2) and subjects provided written informed consent.

### 2.1 Statistical analysis

Since the main study aim was to examine the relationship between different anthropometric variables, notwithstanding the health status of respondents, all analyses are presented for the combined set of 3790 cases and controls, after having confirmed with a Chi-Square test for difference of proportions or T-test for differences of means, that results did not differ by cancer status.

Box-and-whisker plot, mean and standard deviation were used to characterise each silhouette in terms of corresponding weight and BMI, and pants size in terms of waist circumference and WHR. Pearson’s correlation coefficients assessed linear associations, after confirmation of normal distribution. Receiver operating characteristic (ROC) curves were generated to determine how well the silhouettes performed in predicting obesity (BMI ≥ 30 kg/m^2^) versus no-obesity; and how the pants size predicted abdominal obesity (waist circumference ≥ 102 cm). The ROC curve plots the true positive (sensitivity) against the false positive (1-specificity) rate for each scale, while the corresponding area under the curve (AUC) is a global measure of a test’s accuracy (Hoo et al., 2017).

Four different linear regression models were developed from a set of variables easily reported in epidemiological surveys (age, pants size, silhouettes, and weight at the time of the interview). These were compared to identify which predicts best the professionally-measured waist circumference at interview. To build prediction and validation models, we used 10-fold cross-validation models. The data set was divided in 10 random subsets and the holdout method was repeated 10 times. Each time, one of the subsets was used as the test set, and the others were combined to form the training set. Then, average statistics were compiled and compared to test the prediction model (Stone, 1974, Hawkins et al., 2003). Model 0 included age only and served as the basis for comparison. Model 1 added pants size (based on the US chart) to the previous model. Model 2 added the silhouette, while Model 3 added weight (in kg). Silhouettes 1–2 and silhouettes 8–9 were merged to account for small numbers of extreme values in these regressions. Similar models were developed, this time using WHR as the dependent variable. Residuals for normality were verified and the goodness-of-fit was assessed using coefficients of determination (R^2^). Akaike information criteria (AIC, computed as [-2 log likelihood + 2 × number of parameters estimated in the model]) were compared across models (Burnham and Anderson, 2002).

To test the validity of our selected model in predicting measured waist circumference, we compared both predicted and measured waist circumference means with a paired *t* test. We then calculated the intraclass correlation coefficient (ICC) and examined Pearson’s correlation to measure the association between predicted and measured waist circumference values. Finally, a Bland-Altman plot, which shows the difference between the two paired variables (predicted and measured), and the average of these measures, was used to evaluate whether the agreement between the paired variables was related to the waist measurement. Towards this, 95% of the data points should lay within ± 2 standard deviation of the mean difference (Altman and Bland, 1983, Giavarina, 2015). The validity of the best model to predict measured WHR was assessed analogously. All analyses were performed using SAS software (version 9.4; SAS Institute Inc., Cary, NC, USA).

## 3 Results

Selected characteristics of study subjects are presented in Table 1. Subjects were 64 years of age, on average, and mostly of European ancestry. About 23% of men had an educational level below high school. The number of missing values for the different anthropometric indicators across age points for self-reported variables were quite low. Waist and hip circumference measurements were more often unavailable, mostly reflecting interviews taking place in public spaces or the physical condition of the participants (Supplementary Table A.3).

**Table 1:** Selected characteristics of study population from PROtEus, Montreal, Canada, 2005–2012

Variables	*n* = 3790		
Age at recruitment, mean ± IQR^a^	64.1	± 9.0	
Educational level, *n* (%)			
Primary	842	(22.2)	
Secondary / College	1767	(46.6)	
University	1177	(31.1)	
Missing	4	(<0.1)	
Annual family income in $CAN, *n* (%)			
<30 000	944	(24.9)	
30 000–79 999	1700	(44.9)	
>80 000	841	(22.2)	
Other (prefers not to respond, does not know)	305	(8.0)	
Ancestry, *n* (%)			
European	3264	(86.7)	
African	213	(5.7)	
Asian	94	(2.5)	
Other	193	(5.1)	
Do not know	26	(<0.1)	
Alcohol consumption in drink-years (ever users), mean ± IQR	82.5	± 80.8	
Smoking in pack-years (ever smokers), mean ± IQR	25.0	± 28.6	
Silhouette at interview, *n* (%)			
1–4	1027	(27.1)	
5	1562	(41.2)	
6–9	1199	(31.7)	
Body mass index at interview in kg/m^2^, *n* (%)			
Normal (<25)	1195	(31.6)	
Overweight (25–29.9)	1808	(47.9)	
Obese (≥30)	774	(20.5)	
Waist circumference in cm, *n* (%)			
<94	1344	(37.5)	
94–101.9	804	(22.4)	
≥102	1435	(40.1)	
Hip circumference in cm, mean ± IQR	99.1	± 16.6	
Pant size (US chart), mean ± IQR	36.3	± 4.0	

a IQR, interquartile range

Fig. 2a, Fig. 2b depict the correspondence between silhouettes, BMI and weight using box-and-whisker plots. From silhouette 1 to 9, the median and interquartile range (IQR) increased gradually. For most silhouettes, median values for BMI and weight were above those of the previous silhouettes’ 75th percentile, showing a monotonic relationship between the variables. Corresponding data for each plot are presented in Supplementary Table A.4.

**Fig. 2a:**
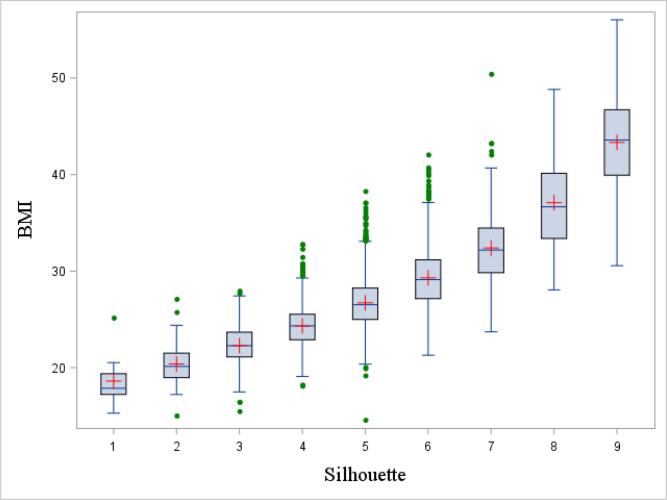
Box-and-whisker plot of the distribution of body mass index (BMI) by silhouettes, at the time of the interview. The bottom and top edge of the box represent the first and third quartile (interquartile range); the line inside the box represents the median; the red cross represents the mean; the ends of the bottom and top whiskers represent the upper and lower adjacent values; and the green filled dots represent outliers. BMI, body mass index. (For interpretation of the references to colour in this figure legend, the reader is referred to the web version of this article.)

**Fig. 2b:**
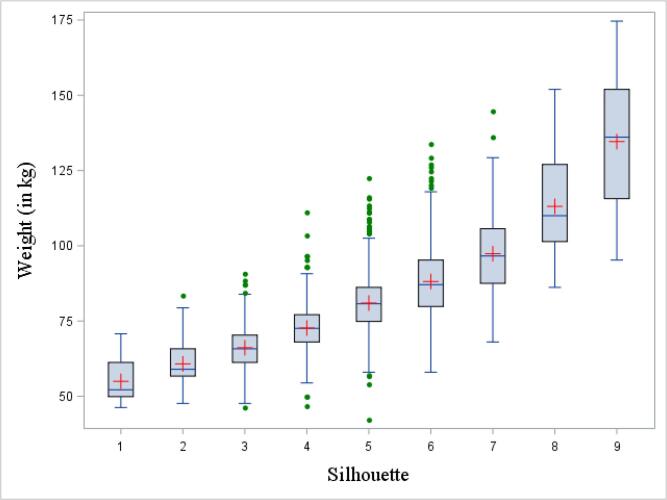
Box-and-whisker plot of the distribution of weight by silhouettes, at the time of the interview. The bottom and top edge of the box represent the first and third quartile (interquartile range); the line inside the box represents the median; the red cross represents the mean; the ends of the bottom and top whiskers represent the upper and lower adjacent values; and the green filled dots represent outliers. (For interpretation of the references to colour in this figure legend, the reader is referred to the web version of this article.)

Fig. 3a, Fig. 3b shows the mean weight (a) and BMI (b), for each silhouette across the five age points. Mean BMIs for silhouettes 1 to 8 were similar, at any given age. For silhouette 9, the mean BMI was more variable between age points (43.3 to 56.5 kg/m^2^), based on small numbers. Supplementary Table A.5 presents the variations around the mean weight and BMI for each silhouette over time. Mean values tended to be slightly lower and more variable at age 20 and data were more scattered for larger silhouettes.

**Fig. 3a:**
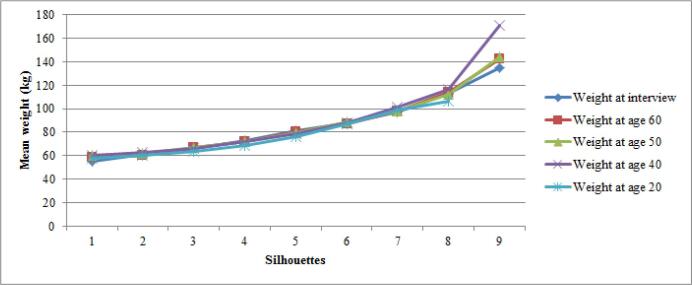
Mean weight for each silhouette at five different age points.

**Fig. 3b:**
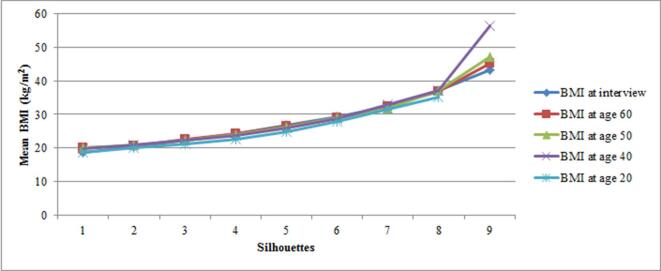
Mean BMI for each silhouette at five different age points.

Fig. 4a, Fig. 4b present the correlation between each pants size, and the mean measured waist circumference and WHR, respectively. Except for pants size 53 and above, the mean waist circumference increased steadily, in a linear fashion, with each increment of pants size. The association between pants size and its corresponding WHR was less well defined, with scattered values at the extremes.

**Fig. 4a:**
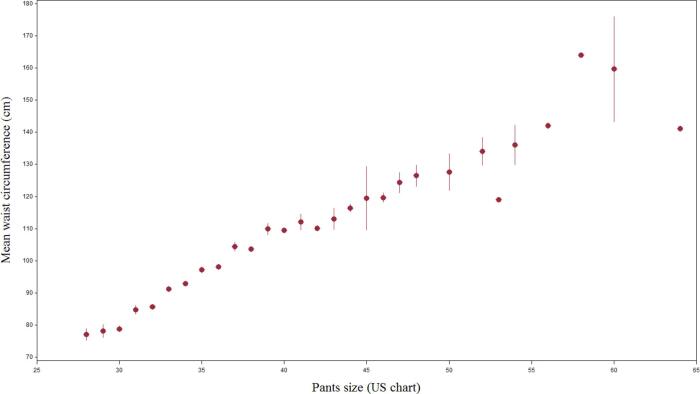
Mean waist circumference (in cm) corresponding to each pants size (US chart), at the time of interview. Vertical lines represent standard deviation.

**Fig. 4b:**
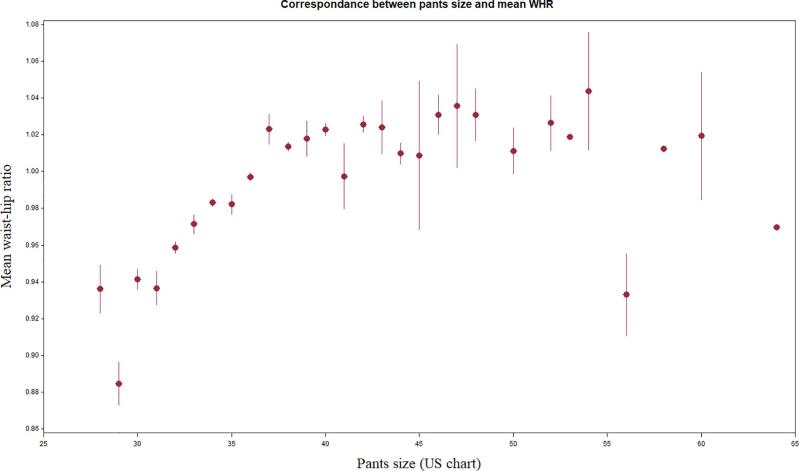
Mean waist-hip ratio corresponding to each pants size (US chart), at the time of interview. Vertical lines represent standard deviation.

Pearson correlation coefficients between anthropometric variables across age points are shown in Table 2. Correlations between silhouettes and BMI ranged between 0.59 and 0.73, with the lowest values observed at ages 20 and 40. Correlations between silhouettes and weight were somewhat lower. Finally, generally high correlations were found between anthropometric indicators at the time of the interview, most notably between waist and hip circumferences (0.90), BMI and silhouette (0.73), weight and BMI (0.89), and weight and silhouette (0.68). The WHR was either poorly or not correlated with the other variables.

**Table 2:** Pearson correlation coefficient between the various anthropometric indicators and across different age points

Age points							
		At age 60	At age 50	At age 40	At age 20		
Silhouette and	Weight	0.68	0.68	0.65	0.56		
	BMI^a^	0.71	0.70	0.68	0.59		
At interview							
	Waistcircumference	Hipcircumference	WHR^b^	Silhouette	Weight	BMI	Pants size
Waist circumference	1.00	0.90	0.45	0.73	0.72	0.72	0.69
Hip circumference		1.00	0.01	0.61	0.61	0.59	0.61
WHR			1.00	0.41	0.39	0.44	0.33
Silhouette				1.00	0.68	0.73	0.69
Weight					1.00	0.89	0.81
BMI						1.00	0.79
Pants size							1.00

a BMI, body mass index

b WHR, waist-hip ratio

The ability to use the silhouettes as a surrogate for BMI in assessing obesity, and then pants size for waist circumference, was tested with ROC curves (Fig. 5a, Fig. 5b). The AUCs for these graphs indicate that the silhouette scale performed well in assessing obesity (AUC = 0.84), so did the pants size in assessing abdominal obesity (AUC = 0.80). For overall obesity, the sensitivity and specificity seemed to be optimal using the 6th silhouette as a cut-off, while for abdominal obesity, pants size of 36 was best.

**Fig. 5a:**
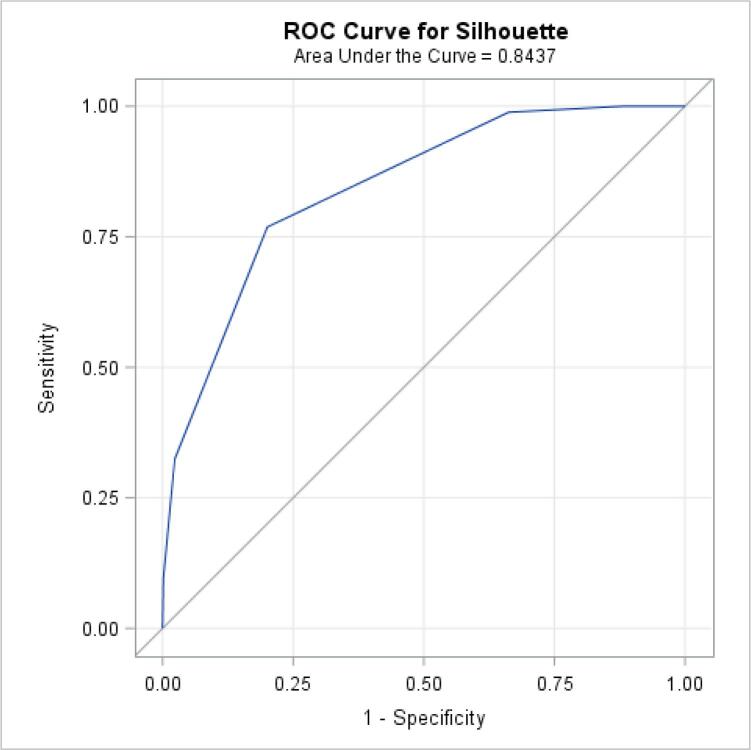
Receiver operating characteristic (ROC) curve for identifying obese subjects (body mass index ≥ 30 kg/m^2^), from the silhouette scale. The blue curved line represents the ROC curve, the diagonal grey line represents the reference line of no discrimination.

**Fig. 5b:**
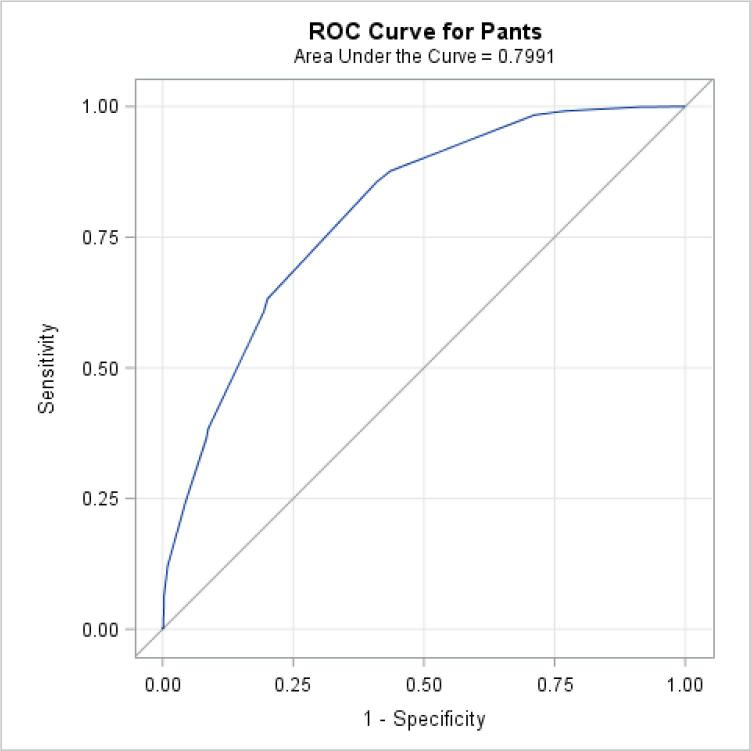
Receiver operating characteristic (ROC) curve for identifying obese subjects (waist circumference ≥ 102 cm), from the pants size. The blue curved line represents the ROC curve, the diagonal grey line represents the reference line of no discrimination.

Table 3 shows the AIC and R^2^ for linear regression Models 0 to 3, adding one anthropometric variable at the time, to model measured waist circumference, and WHR. For a given dataset, a smaller value of the AIC indicates a better fit to the data (Burnham and Anderson, 2002). The AIC decreased markedly with each additional anthropometric variable, especially waist circumference, indicating a better fit when silhouettes and weight were included, than when only using pants size. Concomitantly, the R^2^ increased across models, indicating that the variances for waist circumference, or WHR, were better predicted when incorporating additional anthropometric variables. In Model 3, including age, pants size, silhouette, and weight, the R^2^ was higher for waist circumference than WHR.

**Table 3:** Goodness-of-fit statistics for the different prediction linear regression models for waist and waist-hip ratio, averaged across 10-fold cross-validation samples

Dependant variable	Model 0^a^	Model 1^b^	Model 2^c^	Model 3^d^
Waist circumference				
AIC^e^	15089.3	13015.7	12254.0	12003.6
Δ AIC with model 1			−761.7	
Δ AIC with model 2				−250.4
R^2^	0.00	0.48	0.60	0.64
Waist-hip ratio				
AIC	−16011.3	−16073.1	−16289.1	−16335.7
Δ AIC from Model 1			−216.0	
Δ AIC from Model 2				−46.6
R^2^	0.00	0.11	0.18	0.20

a Model 0 is adjusted for age only

b Model 1 is Model 0 + adjusted for pants size

c Model 2 is Model 1 + adjusted for silhouette

d Model 3 is Model 2 + adjusted for weight

e AIC, Akaike Information Criterion

We used the variables selected from Model 3 to build our prediction model for waist circumference. No differences were observed between mean measured (98.5 cm ± 0.2 SD) and predicted (98.4 cm, ±0.2 SD) waist circumferences. Limits of agreement around the mean of differences, determined from the Bland-Altman plot, showed that most of the mean differences between the two values were within the 95% limits of agreement (Fig. 6a). The ICC between the measured and predicted values was 0.79. However, a relationship was observed between the predicted and measured waist circumferences (*r* = 0.33, p < 0.01), where a positive *r* value represents an overestimation of predicted waist circumference compared with measured waist circumference, for those with a larger waist circumference than the mean.

**Fig. 6a:**
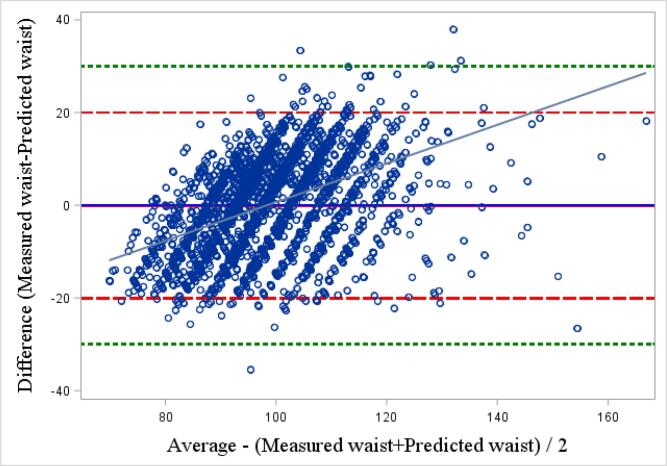
Bland-Altman plot of the difference between predicted and professionally measured waist circumference. The full horizontal blue line represents the mean between the 2 values, larger red dash lines correspond to twice the standard deviation of the mean difference and smaller green dash lines represent 3 times the standard deviation.

No difference was observed between the mean measured and predicted WHR. However, the Bland-Altman plot indicates that more correspondences fell beyond the 95% limit of agreement (±2 standard deviations) and some were even outside of 3 standard deviations (Fig. 6b), as evident from the weak fit of Model 3. The ICC between the two variables was lower than in the model for waist circumference prediction (0.52). A more pronounced relationship was observed between the predicted and mean WHR (*r* = 0.69, p < 0.01), compared to that based on waist circumference.

**Fig. 6b:**
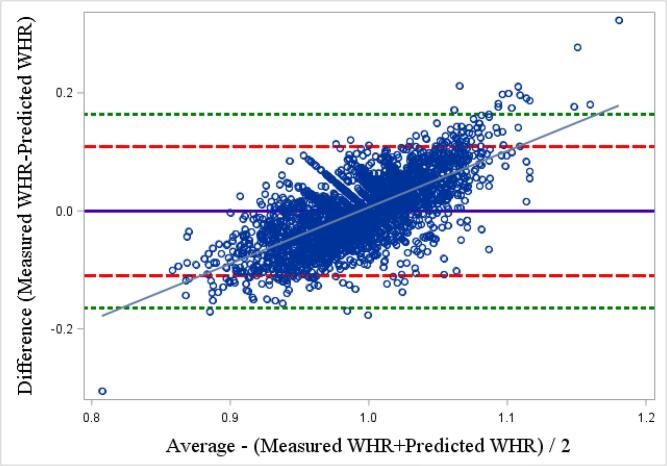
Bland-Altman plot of the difference between predicted and professionally measured waist-hip ratio. The full horizontal blue line represents the mean between the 2 values, larger red dash lines correspond to twice the standard deviation of the mean difference and smaller green dash lines represent 3 times the standard deviation.

## 4 Discussion

We observed that Stunkard’s silhouette scale was closely related to reported BMI and weight, currently or decades in the past among adult males. Moreover, we found that a set of reported variables easily obtained in the context of epidemiological studies such as BMI, silhouettes, and pants size can be used to predict measured abdominal obesity, especially waist circumference, reasonably well.

The observed associations between each reported silhouette and mean BMI were similar to those reported previously among men (Mueller et al., 1985, Bulik et al., 2001), with differences between each drawing representing an increase of about 2 or 3 units of BMI. We also showed that, while the mean BMI and weight for each silhouette increased gradually, the range between the first and third quartile did overlap in most silhouettes. Using the classification of the World Health Organization (World Health Organization 2000), silhouettes 5 and 6 were consistent with overweight or pre-obesity (BMI between 25.00 and 29.99 kg/m^2^). Silhouettes 7, 8 and 9 reflected obesity class I (BMI of 30.00–34.99 kg/m^2^), obesity class II (BMI of 35.00–39.99 kg/m^2^) and obesity class III (BMI ≥ 40.00 kg/m^2^), respectively.

Most previous studies assessing the relationship between Stunkard’s silhouettes and current BMI found moderate to good correlations, but the way information on anthropometric factors was obtained varied across studies. A few studies (Stunkard et al., 1983, Mueller et al., 1985, Must et al., 1993) used BMI derived from professionally-measured weight and height, while our comparison was based on self-reports. However, many studies have shown that the accuracy of self-reported and professionally-measured BMI are comparable (Sorensen and Stunkard, 1993, Connor Gorber et al., 2007, Poston et al., 2014). This is also true for remote recall of weight and silhouette (Must et al., 1993, de Fine Olivarius et al., 1997, Tamakoshi et al., 2003). Our observed correlations were stronger for the time of the interview (r = 0.75) than for the age of 20 years (r = 0.59) and are compatible with other studies, i.e., r = 0.53 for remote recall for the age of 20 years (Must et al., 1993), and r = 0.63 for a 10 years recall (Lønnebotn et al., 2018).

The correlation between professionally-measured waist circumference and pants size observed was 0.66, in accordance with previous findings (0.64 – 0.87), either using self-reported (Hughes et al., 2009, Battram et al., 2011, Moy et al., 2018) or professionally-measured circumference (Han et al., 2005). We also observed a strong correlation between waist or hip circumference and the other anthropometric variables (weight, BMI and silhouettes), similarly to others (Fowke et al., 2012, Guerrios-Rivera et al., 2017), where *ρ* was superior to 0.7. This suggests that these anthropometric variables are often interlinked. The null correlation found between WHR and hip circumference was expected; when a ratio variable is correlated with one of the ratio variable components, it is affected by negative bias (Schuessler, 1974, Macmillan and Daft, 1980). Correlation measures the linear association between variables. To evaluate the accuracy of the silhouette scale and pants size to correctly assess obesity, we computed ROC curves and area under the curve. The corresponding analyses yielded high AUC values similar to those reported in previous studies (Bulik et al., 2001, Kaufer-Horwitz et al., 2006, Dratva et al., 2016). Our cut-off point for identifying obese men (based on BMI), the sixth silhouette, also agrees with results reported in these previous articles.

Another important contribution of this work is the development of a prediction model for measured waist circumference and for WHR based on a set of simple variables characterized by high reporting rates (pants size, silhouette, and weight). The regression models we developed showed a good fit, particularly for predicting waist circumference. While the mean of the predicted waist circumference was nearly identical to the measured value, we observed a pattern of overestimation of predicted values for men with a larger measured waist circumference. However, extreme values were based on few individuals. In addition, in prediction analyses, differences were associated with the average, while there were substantial individual variations. Battram et al. (Battram et al., 2011) observed a non-significant positive correlation between predicted and measured variables (*r* = 0.24). Unlike ours, their equation used an indicator for fitness of pants. A similar prediction model of BMI, with the use of the silhouette scale and various social and health related variables also observed a good fit (R^2^ = 0.52) (Dratva et al., 2016). However, some of these models were built using self-reported values of circumferences, introducing some bias if these values were too distant than their real counterpart (Keith et al., 2011). Given that waist circumference has recently been shown to be a stronger predictor than BMI for several health outcomes (Rhee et al., 2018), research into the development of alternatives to professionally-based assessment of abdominal obesity that can be easily used in large-scale investigations is highly valuable.

Differential reporting between groups is always possible in case-control studies. However, there was no evidence that this was operating in our study for the variables of interest, as results did not differ according to case/control status (not shown), thus justifying combined analyses. Moreover, it is unlikely that reporting would have differed for some of the anthropometric constructs and not others.

The use of self-reports, entailing misclassification, precludes us from concluding on validity about alternate indicators of overall obesity, unlike for those of abdominal obesity which relied on measurements. Nevertheless, several validation studies have shown that when prospective measurement cannot be obtained, the retrospective recall of weight and obesity in the remote past is reliable and valid (Stevens et al., 1990, Must et al., 1993, de Fine Olivarius et al., 1997, Gunnell et al., 2000). Stevens et al. found a strong correlation (r = 0.82) between a 28 year earlier recalled and measured weight, while Must et al. found a moderate correlation (r = 0.53) between a recalled silhouette 50 years earlier and measured BMI. In our study, correlations between silhouettes and reported BMI were remarkably stable across adulthood. However, recalls were repeated over time and were not independent; subjects might have tended to readily associate, for example, little changes in weight with little changes in silhouettes.

Misclassification of predicted waist circumference based on clothing size may have occurred for different reasons. Some people prefer looser fit, and wear their pants above or below the umbilicus based on personal preferences or fashion standards. While pants size would be expected to correspond to the waist circumference, the degree of correspondence can vary greatly between brands (Kinley 2003). Based on our data, pants size was on average 2 inches narrower than the measured waist circumference (data not shown). Differences among manufacturers have been reported and sizing systems differ between countries. Direct association between clothing size and waist measurements should be interpreted with caution (Kinley, 2003, Hughes et al., 2009). Despite these issues, a study showed that only 3% of participants reported a clothing size different from the clothing labels (Han et al., 2005).

Our study presents several strengths. Data were collected by trained interviewers as part of face-to-face interviews. Information was elicited on different anthropometric variables over the entire adulthood period, each representing different aspects of body size. Waist and hip circumferences were measured according to a strict protocol. Based on the lower number of missing responses, it appears that silhouettes and pants size are more easily reported than other anthropometric variables. However, the evidence from large studies investigating their associations with commonly used variables such as BMI and waist circumference is scarce. Given the distribution of participants across BMI and waist circumference categories observed, it appears that the results could be generalizable to older community-dwelling men.

## 5 Conclusion

In summary, Stunkard’s silhouette scale proved to be an easy-to-administer tool that was closely related to reported BMI and weight among adult males, both currently and in the past. It appropriately ranked individuals according to commonly used obesity categories. We observed that a model including age, reported pants size, silhouette and weight could reasonably predict current abdominal obesity, especially when using waist circumference as the indicator. Ideally, anthropometric variables should be measured directly; however, for logistic and monetary reasons, this is often impossible. Approaches that can be used as alternatives to measurements, especially when earlier exposures are thought to be etiologically relevant, need to be further evaluated.

## CRediT authorship contribution statement

**Eric Vallières:** Conceptualization, Methodology, Software, Validation, Formal analysis, Writing - original draft, Visualization. **Marie-Hélène Roy-Gagnon:** Methodology, Writing - review & editing. **Marie-Élise Parent:** Conceptualization, Investigation, Resources, Data curation, Writing - review & editing, Supervision, Project administration, Funding acquisition.

## Declaration of Competing Interest

The authors declare that they have no known competing financial interests or personal relationships that could have appeared to influence the work reported in this paper.
