# Supplementary material for: Body shape and pants size as surrogate measures of obesity among males in epidemiologic studies
Source: Prev Med Rep. 2020 Jul 13;20:101167. doi: 10.1016/j.pmedr.2020.101167 (PMC7479209; doi:10.1016/j.pmedr.2020.101167)
Supplement: Supplementary data 1 [file mmc1.docx]

**Supplementary material**

Supplementary Table A.1. International pants size conversion chart^a^, for men

| USA | France | Germany | Italy | Spain | UK | Russia |
| --- | --- | --- | --- | --- | --- | --- |
| 28-29 | 38/40 | 28/29 | 38/40 | 71 | 28/29 | 44/46 |
| 30-31 | 40/42 | 30/31 | 40/42 | 76 | 30/31 | 46/48 |
| 32-33 | 42/44 | 32/33 | 42/44 | 81 | 32/33 | 48/50 |
| 34 | 44 | 34 | 44 | 87 | 34 | 50 |
| 36 | 46 | 36 | 46 | 92 | 36 | 52 |
| 38 | 48 | 38 | 48 | 97 | 38 | 54 |
| 40 | 50 | 40 | 50 | 102 | 40 | 54/56 |
| 42 | 52 | 42 | 52 | 107 | 42 | 56/58 |

^a^Adapted from Levi’s^®^ website (<https://www.levi.com/US/en_US/cms/sizeguide>)

Supplementary Table A.2. List of participating institutions

| **Hospital Name** |
| --- |
| Notre-Dame Hospital |
| St-Luc Hospital |
| Hôtel-Dieu de Montréal Hospital |
| Maisonneuve-Rosemont Hospital |
| Jean-Talon Hospital |
| Charles-Lemoyne Hospital |
| Centre hospitalier Fleury |

Supplementary Table A.3. Number and percentage of missing values for the various anthropometric indicators^a^

| **Variables** | **At interview** | | **At age 60** | | **At age 50** | | **At age 40** | | **At age 20** | |
| --- | --- | --- | --- | --- | --- | --- | --- | --- | --- | --- |
|  | **n** | **%** | **n** | **%** | **n** | **%** | **n** | **%** | **n** | **%** |
| **Self-reported** |  |  |  |  |  |  |  |  |  |  |
| Silhouette | 2 | 0.1 | 5 | 0.2 | 8 | 0.2 | 7 | 0.2 | 7 | 0.2 |
| Weight (kg) | 12 | 0.3 | 18 | 0.6 | 35 | 1.0 | 51 | 1.4 | 137 | 3.6 |
| Height (m) | 17 | 0.4 |  |  |  |  |  |  | 22 | 0.6 |
| Body mass index (kg/m^2^) | 13 | 0.3 | 20 | 0.7 | 36 | 1.0 | 52 | 1.4 | 139 | 3.7 |
| Pants size (US chart) | 65 | 1.7 | 73 | 2.6 | 111 | 3.0 | 152 | 4.0 | 329 | 8.7 |
| **Measured by interviewer** |  |  |  |  |  |  |  |  |  |  |
| Waist circumference (cm) | 207 | 5.5 |  |  |  |  |  |  |  |  |
| Hip circumference (cm) | 210 | 5.5 |  |  |  |  |  |  |  |  |
| Waist-hip ratio | 212 | 5.6 |  |  |  |  |  |  |  |  |
| **Total*** | 3790 |  | 2844 |  | 3703 |  | 3790 |  | 3790 |  |

^a^ Totals varied, as some subjects did not reach age 50 or 60 at the time of the interview.

Supplementary Table A.4. Body mass index and weight (n, mean, 25^th^ and 75^th^ percentile), by Stunkard’s silhouette

|  | Silhouette | | | | | | | | |
| --- | --- | --- | --- | --- | --- | --- | --- | --- | --- |
|  | 1 | 2 | 3 | 4 | 5 | 6 | 7 | 8 | 9 |
| **Body mass index** |  |  |  |  |  |  |  |  |  |
| n | 11 | 68 | 273 | 672 | 1558 | 874 | 242 | 60 | 18 |
| Median | 17.9 | 20.1 | 22.3 | 24.3 | 26.5 | 29.1 | 32.2 | 36.7 | 43.6 |
| 25^th^ percentile | 17.2 | 19.0 | 21.1 | 22.9 | 25.0 | 27.1 | 29.8 | 33.4 | 39.9 |
| 75^th^ percentile | 19.4 | 21.5 | 23.7 | 25.5 | 28.2 | 31.2 | 34.5 | 40.1 | 46.7 |
| **Weight** |  |  |  |  |  |  |  |  |  |
| n | 11 | 68 | 273 | 672 | 1557 | 874 | 243 | 60 | 18 |
| Median | 52.2 | 59.0 | 65.8 | 24.3 | 80.7 | 87.1 | 96.6 | 110.0 | 136.1 |
| 25^th^ percentile | 49.9 | 56.7 | 61.2 | 22.9 | 74.8 | 79.8 | 87.5 | 101.4 | 115.7 |
| 75^th^ percentile | 61.2 | 65.8 | 70.3 | 25.5 | 86.2 | 95.3 | 105.7 | 127.0 | 152.0 |

Supplementary Table A.5. Mean weight and body mass index (BMI), with associated standard deviations (sd), corresponding to each silhouette at 5 different age points

|  | Silhouettes | | | | | | | | | | | | | | | | | |
| --- | --- | --- | --- | --- | --- | --- | --- | --- | --- | --- | --- | --- | --- | --- | --- | --- | --- | --- |
|  | 1 | | 2 | | 3 | | 4 | | 5 | | 6 | | 7 | | 8 | | 9 | |
|  | Mean | sd^a^ | Mean | sd | Mean | sd | Mean | sd | Mean | sd | Mean | sd | Mean | sd | Mean | sd | Mean | sd |
| Weight (kg) |  |  |  |  |  |  |  |  |  |  |  |  |  |  |  |  |  |  |
| At interview | 55.0 | 7.3 | 60.8 | 7.4 | 66.1 | 7.8 | 72.7 | 7.6 | 81.0 | 9.7 | 88.1 | 11.6 | 97.4 | 12.7 | 113.1 | 16.1 | 134.6 | 23.3 |
| At age 60 | 58.7 | 8.0 | 60.6 | 7.8 | 66.9 | 7.1 | 72.3 | 7.6 | 80.1 | 9.5 | 87.2 | 10.9 | 97.8 | 14.3 | 114.0 | 15.5 | 142.5 | 22.7 |
| At age 50 | 60.3 | 7.9 | 62.0 | 7.7 | 66.5 | 6.9 | 72.2 | 7.6 | 79.8 | 9.2 | 88.9 | 11.5 | 98.2 | 12.5 | 112.4 | 17.9 | 144.4 | 24.5 |
| At age 40 | 59.8 | 7.3 | 62.7 | 7.2 | 66.3 | 7.0 | 71.6 | 8.0 | 78.8 | 9.4 | 88.3 | 11.6 | 101.2 | 13.8 | 116.2 | 24.6 | 170.9 | 37.7 |
| At age 20 | 57.3 | 6.9 | 60.3 | 7.1 | 63.9 | 7.3 | 68.7 | 8.8 | 75.9 | 10.2 | 87.2 | 11.9 | 98.4 | 17.9 | 106.0 | 14.7 | - | - |
| BMI (kg/m^2^) |  |  |  |  |  |  |  |  |  |  |  |  |  |  |  |  |  |  |
| At interview | 18.6 | 2.6 | 20.4 | 2.2 | 22.3 | 2.1 | 24.3 | 2.2 | 26.7 | 2.7 | 29.3 | 3.3 | 32.4 | 3.5 | 37.1 | 4.8 | 43.3 | 6.4 |
| At age 60 | 20.0 | 2.5 | 20.6 | 2.3 | 22.7 | 2.1 | 24.3 | 2.2 | 26.6 | 2.7 | 29.1 | 3.1 | 32.2 | 4.0 | 37.0 | 5.0 | 45.3 | 7.0 |
| At age 50 | 20.2 | 2.7 | 21.0 | 2.5 | 22.4 | 1.9 | 24 | 2.2 | 26.3 | 2.5 | 29.1 | 3.1 | 31.9 | 3.5 | 37.2 | 6.1 | 47.1 | 9.8 |
| At age 40 | 19.9 | 2.5 | 21.0 | 2.2 | 22.2 | 1.9 | 23.8 | 2.3 | 25.9 | 2.6 | 28.7 | 3.0 | 32.8 | 4.0 | 37.1 | 6.4 | 56.5 | 8.8 |
| At age 20 | 18.6 | 1.9 | 20.0 | 2.0 | 21.3 | 2.1 | 22.6 | 2.4 | 24.8 | 2.9 | 27.8 | 3.5 | 31.7 | 5.1 | 35.3 | 6.8 | - | - |
